# The prognostic value of serum levels of a proliferation-inducing ligand (APRIL) in treatment-naïve patients with chronic lymphocytic leukemia

**DOI:** 10.3906/sag-2004-282

**Published:** 2021-02-26

**Authors:** Sinem Nihal ESATOĞLU, Dilek KESKİN, Ahmet Emre EŞKAZAN, Tuğrul ELVERDİ, Ayşe SALİHOĞLU, Muhlis Cem AR, Şeniz ÖNGÖREN, Zafer BAŞLAR, Yıldız AYDIN, Hafize UZUN, Teoman SOYSAL

**Affiliations:** 1 Department of Internal Medicine, Section of Rheumatology, Cerrahpaşa Medical Faculty, İstanbul University-Cerrahpaşa, İstanbul Turkey; 2 Department of Internal Medicine, Section of Hematology, Cerrahpaşa Medical Faculty, İstanbul University-Cerrahpaşa, İstanbul Turkey; 3 Department of Biochemistry, Cerrahpaşa Medical Faculty, İstanbul University-Cerrahpaşa, İstanbul Turkey

**Keywords:** Chronic lymphocytic leukemia, treatment, survival, prognosis, chemotherapy

## Abstract

**Background/aim:**

A proliferation-inducing ligand (APRIL) has been investigated as a prognostic marker in chronic lymphocytic leukemia (CLL) patients. However, there is no cut-off level for serum APRIL (sAPRIL) levels that predict time to treatment in CLL patients.

**Materials and methods:**

Between May and December 2012, 94 consecutive CLL patients and 25 healthy controls were assessed. sAPRIL levels were measured by ELISA. Demographic data and prognostic markers were obtained from the patients’ files. Treatment-naïve patients were followed up for 6.5 years for any treatment need.

**Results:**

Patients were divided into 3 groups: Treatment-naïve (n = 47), chemotherapy receiving (n = 25), and those who had received chemotherapy previously (n = 22). There was no difference in median sAPRIL levels of patients who were receiving chemotherapy at the sampling time and the healthy controls, which indicates that sAPRIL levels might be influenced by treatment. For treatment-naïve patients, the best cut-off in predicting time to treatment was found at the sAPRIL level of 2.04 ng/mL, with 78% sensitivity and 63% specificity. Time to treatment was significantly earlier in the APRIL high group (n = 27) than in the APRIL low group (n = 20) (P = 0.010, log-rank test).

**Conclusion:**

sAPRIL, a simple, promising blood test which can be measured by ELISA, will likely obtain a place in the wide range of prognostic markers in CLL. Prospective large-scale studies are required to validate and confirm the feasibility of the proposed cut-off level of 2.04 ng/mL as a predictor of time to treatment in treatment-naïve CLL patients.

## 1. Introduction

Chronic lymphocytic leukemia (CLL) is a progressive malignant disease characterized by the accumulation of monoclonal lymphocytes in peripheral blood, bone marrow, and lymphoid tissues. Immunophenotypic analysis by flow cytometry reveals CD5, CD19, CD20, and CD23 expression in B cells [1]. Treatment is required in cases with active disease, which is defined by the following conditions: B symptoms, progressive splenomegaly, hepatomegaly, lymphadenopathy or lymphocytosis, evidence of bone marrow failure that is not caused by autoimmune phenomena and organomegaly, and autoimmune phenomena refractory to conventional therapy [2]. Unlike other types of leukemia, treatment is usually deferred until advanced stages of disease [2]. There are no treatment options for cure except for allogeneic bone marrow transplantation. 

There are 2 clinical staging systems in CLL: the Rai [3] and the Binet systems [4]. Although they are easy to use and are widely used for determining prognosis, both staging systems fall short in identifying a proportion of CLL patients who have early-stage disease but are at high risk for faster progression. Therefore, there has been a considerable amount of research to identify patients at risk of progression [5]. Several complementary parameters have been suggested to improve the prediction potential of prognostic scoring systems, such as shortened lymphocyte doubling time (LDT), beta2-microglobulin (β2M), lactate dehydrogenase (LDH), thymidine kinase, CD49d levels, CD38, and zeta-chain–associated protein kinase 70 (ZAP70) expression. Furthermore, cytogenetic abnormalities including deletions in chromosomes 11, 13, and 17, mutations in the immunoglobulin heavy chain gene variable region (IGHV) and TP53 and several mutated genes that are involved in DNA damage, Notch signaling, inflammatory pathways, and cytokine signaling have been identified as prognostic markers [5,6]. Since none of the aforementioned parameters can identify all patients at high risk and the routine clinical use of most of them is limited, there is an unmet need for a better marker for prognosis. 

A proliferation-inducing ligand (APRIL) belongs to the family of tumor necrosis factor ligands and has been known to be associated with B cell proliferation and survival [7]. It plays different roles at various stages of B-cell ontogeny. There have been some observations that APRIL might play a role in the pathogenesis of CLL. First, CLL cells have been shown to express APRIL, which was held responsible for the resistance to apoptosis of CLL cells [7]. Second, nurse-like cells that are a part of the CLL microenvironment have been shown to express APRIL [8]. In addition to those, APRIL transgenic mice are prone to develop B-cell–associated lymphoid tumors [9]. Moreover, serum APRIL (sAPRIL) levels have been found to be associated with a shorter treatment-free interval in newly diagnosed CLL patients [10,11]. However, there are conflicting data regarding its prognostic role in survival [10,12,13]. Finally, there is no data on whether sAPRIL levels are still a useful prognostic tool during the course of the disease, and how they vary according to treatment.

The aim of the present study was (1) to compare sAPRIL levels of CLL patients with those of age- and sex-matched healthy subjects, (2) to investigate the relationship between sAPRIL levels and other common prognostic factors, (3) to find out whether sAPRIL levels are influenced by treatment, (4) to determine whether sAPRIL levels can predict time to treatment in the setting of a prospective observational study over a time span of up to 6.5 years, and (5) to identify a cut-off level for prediction of time to treatment.

## 2. Materials and methods

Between May and December 2012, venous blood samples were drawn from 104 consecutive CLL patients and 25 age- and sex-matched healthy controls. Treatment-naïve patients have been followed up with for 6.5 years for any treatment requirements and survival. CLL was diagnosed according to the National Cancer Institute Working Group criteria [14]. The study was supported by an unrestricted grant by Scientific Research Projects Coordination Unit of İstanbul University (Project No.: 19694) and was approved by the Ethics Committee of the Cerrahpaşa Medical Faculty (43458/2011). Written informed consent was obtained from all of the patients and healthy controls. This study was conducted in accordance with the 1964 Helsinki Declaration and its later amendments or comparable ethical standards.

Data at initial presentation of the patients were reviewed from the medical records for the following parameters: demographic features, the presence of organomegaly and peripheral lymphadenopathy, complete blood count, LDH and β2M levels, ZAP-70 positivity, the percentage of CD38+ cells in the flow cytometry, and time to treatment. The cut-off levels for CD38+ positivity and β2M were 30% and 2 mg/L, respectively. The cut-off of del17p performed by fluorescent in situ hybridization (FISH) in our laboratory was 10%.

Venous blood samples were collected in anticoagulant-free tubes without venous stasis after 12 h of overnight fasting, and centrifuged immediately (3000 g) for 10 min at +4 °C. The serum was stored at –80 °C until the time of assay. sAPRIL levels were measured in duplicate aliquots, using a human enzyme-linked immunosorbent assay (ELISA) test according to the manufacturer’s instructions (Bender MedSystems, Vienna, Austria). The coefficients of intra- and interassay variations were 4.1% (n = 10) and 7.2% (n = 10), respectively.

Statistical analysis was done using SPSS 17.0. Categorical variables were compared by chi-square test. Continuous variables were compared with Student’s t-test when data were parametric and with the Mann–Whitney U test when data were nonparametric. Spearman’s correlation test was used to assess the correlation between measures. Summary receiver operating characteristic (ROC) curve and log-rank test were used to calculate whether sAPRIL levels predicted time to treatment in treatment-naïve CLL patients. Statistical significance was considered at the two-tailed 0.05 level.

## 3. Results

Of 104 patients, 10 were excluded from the study due to hemolyzed blood samples (3 patients), Richter transformation at the time of recruitment (3 patients), and inadequacy of medical records (4 patients). Overall, samples from 94 CLL patients and 25 healthy donors were eligible for the final analysis.

**Figure 1:**
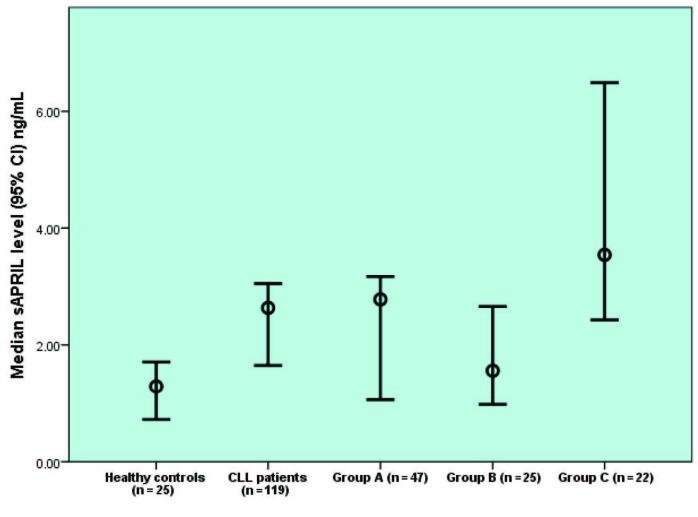
Median sAPRIL levels of healthy controls, CLL patients, treatment-naïve patients (group A), patients receiving chemotherapy (group B), and patients who were received chemotherapy previously (group C).

Median sAPRIL levels of CLL patients were found to be significantly higher than those of 25 healthy donors (2.63 ng/mL, IQR: 0.97–3.75 vs 1.29 ng/mL, IQR: 0.58–2.19, respectively; P = 0.006) (Figure 1). At the sampling time, 47 patients were treatment-naïve, 25 patients were actively receiving chemotherapy, and 22 patients had received chemotherapy previously and had been treatment-free for ≥3 months. The median sAPRIL levels of 47 treatment-naïve and 22 treated patients were significantly higher than those of the healthy controls (2.78 ng/mL, IQR: 0.61–3.78 vs. 1.29 ng/mL, IQR: 0.58–2.19; P = 0.028 and 3.54 ng/mL, IQR: 2.23–6.51 vs. 1.29 ng/mL, IQR: 0.58–2.19); P <0.001, respectively). However, the median sAPRIL levels of 25 patients actively receiving chemotherapy and 25 healthy controls were not different (1.56 ng/mL, IQR: 0.84–2.99 vs. 1.29 ng/mL, IQR: 0.58/–2.19; P = 0.295) (Figure 1). Although we did not measure sAPRIL levels prior to or after chemotherapy in each patient, obtained data showing no difference in median sAPRIL levels of patients who were receiving chemotherapy and the controls made us think that sAPRIL levels might be influenced by treatment. We then extended the study to follow-up with treatment-naïve patients to delineate the prognostic role of sAPRIL in these patients. 

**Treatment-naïve patients (group A)**

There were 47 (M/F: 30/17) treatment-naïve CLL patients. ZAP-70 results were available in 9 patients, with 2 of them being positive. 

sAPRIL levels were found to be negatively correlated with haemoglobin levels (r = –0.298; P = 0.037) and platelet counts (r = –0.321; P = 0.025). There were no correlations with prognostic indicators such as age (r = 0.069; P = 0.64), Rai (r = 0.151; P = 0.31) and Binet stages (r = 0.171; P = 0.24), lymphocyte counts (r = 0.039; P = 0.79), β2M (r = 0.121; P = 0.18), or CD38 levels (r = 0.037; P = 0.85). The median sAPRIL levels were not different among the patients who had high or normal LDH, CD38, and β2M levels (data not shown). Clonal abnormality was not evaluated due to the low number of detected cases (5 patients).

The median follow-up times of the patients since diagnosis and serum sampling were 114 months (IQR: 90-138) and 78 months, respectively. Among the 47 patients, 23 received chemotherapy due to progressive disease. The median time from sampling to treatment in these 23 patients was 37 months (IQR: 17–47). Seven patients died during the follow-up period, and 5 of them received treatment due to progressive CLL and died due to refractory disease and infection after a median follow up of 1.5 years (range: 1–3) following treatment initiation. The remaining 2 treatment-naïve patients died due to cardiovascular disease 3.5 and 4 years after the study entry; at the time of death, they still had not required therapy.

**Figure 2:**
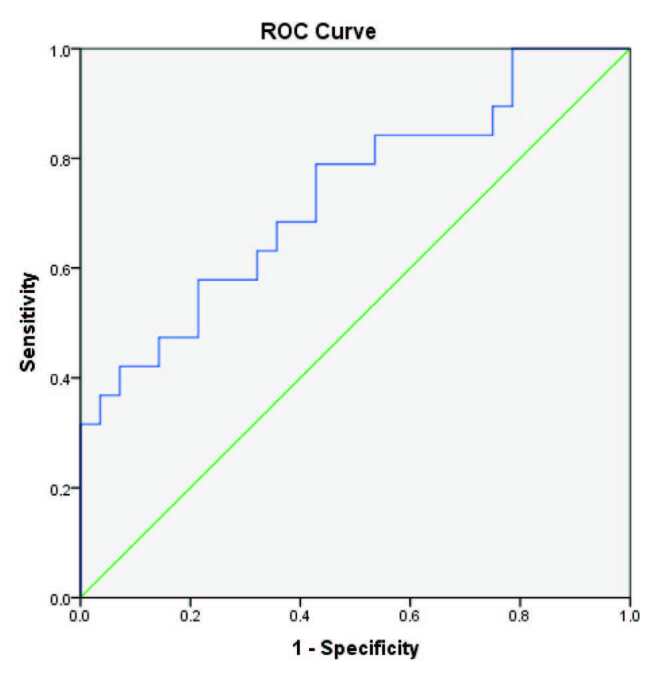
The ROC curve of sAPRIL in predicting time for the treatment.

**Figure 3:**
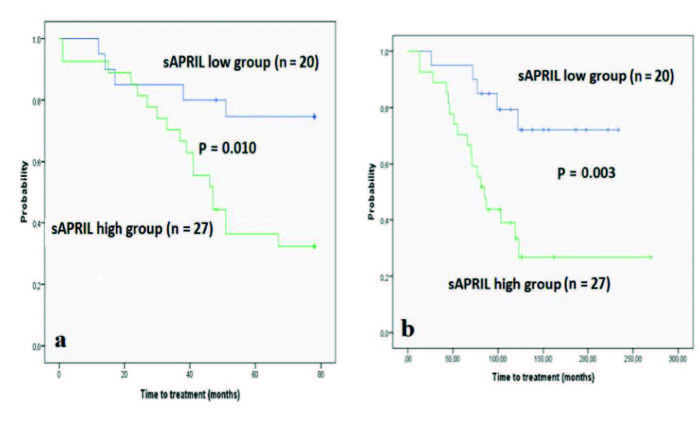
Kaplan-Meier graph shows an earlier time to treatment from sampling (3a) and diagnosis (3b).

The ROC curve of sAPRIL levels in predicting time to treatment showed an area under the curve of 0.75 (Figure 2). The best cut-off in terms of prognostic effectiveness was found at an sAPRIL level of 2.04 ng/mL, with 78% sensitivity and 63% specificity. The patients were divided into 2 groups according to the cut-off level: sAPRIL high (n = 27) and sAPRIL low (n = 20). The 2 groups were similar with respect to demographic data and prognostic factors (Table). In the sAPRIL high group, 18 of the 27 patients received chemotherapy during follow-up whereas only 5 of the 20 patients in the sAPRIL low group required treatment. Time to treatment from sampling (Figure 3a) and diagnosis (Figure 3b) was significantly earlier in the sAPRIL high group than in the sAPRIL low group (P = 0.010, P = 0.003, log-rank test, respectively). Among the 5 patients who had died due to refractory CLL and infection, 4 were in the sAPRIL high group and 1 was in the sAPRIL low group. One patient died in each group due to cardiovascular disease.

**Table:** The demographic and clinical characteristics of sAPRIL high and sAPRIL low group.

	APRIL high group (n = 27)	APRIL low group(n = 20)	P-value
Mean ± SD age at sampling time, years	67 ± 12.4	66.9 ± 10.2	0.98
Mean ± SD age at diagnosis, years	64.3 ± 10.5	61 ± 10.2	0.48
Male, n (%)	19 (70.4)	11 (55)	0.36
Binet stage			
A	22	16	0.89
B	3	3	0.69
C	2	1	0.74
Modified Rai stage			
Low-risk	18	15	0.83
Intermediate-risk	7	3	0.36
High-risk	2	2	0.75
Median (IQR) lymphocyte, mm3	23.700 (11.100-56910)	18.755 (15.112-30300)	0.83
LDH, n (%)	25 (93)	17 (85)	0.4
Median (IQR) β2M (mg/L)	2125 (1680-2862)	1985 (1759-2637)	0.21
High CD38, n/N (%)	0/13	3/17 (18)	0.11
17p deletion, n/N (%)	4/15 (26)	1/9 (11)	0.36
Patients who required chemotherapy during the follow-up, n (%)	18 (67)	5 (25)	0.004

APRIL: a proliferation-inducing ligand; β2M: beta2-microglobulin; LDH: lactate dehydrogenase.

## 4. Discussion

Several studies have been conducted to predict outcomes in patients with CLL since patients having early-stage disease have a variable clinical course. None of the prognostic factors mentioned above can stratify all patients at high risk for progression. Moreover, most of them are not available in routine clinical practice. It would be desirable to have a simple prognostic test. sAPRIL levels are easily measured by ELISA and have been investigated as a prognostic marker in CLL patients. However, previous studies have reported conflicting results with regard to the prognostic role of sAPRIL in terms of overall survival [12,13,10]. Additionally, its prognostic role has been mostly studied in newly diagnosed patients. In the present study, we included consecutive CLL patients. Although our patient population was heterogeneous regarding disease duration, sAPRIL levels again predicted time to treatment in treatment-naïve CLL patients based on an ROC-analysis–defined threshold of 2.04 ng/mL. We also found that sAPRIL levels seem to be influenced by treatment. 

Tecchio and colleagues [10] reported that high sAPRIL levels were associated with an earlier progression in low-risk CLL patients. Ferrer and colleagues [11] also supported this finding and showed that a combined analysis of B-cell activating factor and sAPRIL levels may be more useful to predict disease progression in CLL patients. Our results confirmed those of the previous studies, showing that sAPRIL levels can predict time to treatment in treatment-naïve CLL patients. However, unlike the 2 previous retrospective studies in which the serum samples were collected at the time of diagnosis, our study was cross-sectional and included samples obtained at the diagnosis or during the course of the disease. Despite this heterogeneity in the collection time points of the samples, sAPRIL remained a useful predictor of time to treatment in treatment-naïve CLL patients. This finding is remarkable as it indicates that sAPRIL levels may be used as a prognostic factor independent of time of sample collection. Moreover, unlike in the previous studies, in our hands, the ROC curve provided a cut-off level of 2.04 ng/mL, which clearly differentiated patients who would need treatment. 

Serum [12] and plasma [13] APRIL levels were found to be associated with overall survival in 2 studies, but not in Tecchio’s study [10]. In the study by Planelles et al. [12], the patient group with high sAPRIL levels included more patients with advanced-stage disease when compared to the group with low sAPRIL levels. This might be the reason for the conflicting results with regard to the prognostic potential of sAPRIL levels. On the other hand, the demographic and clinical differences among the sAPRIL high and sAPRIL low groups were not clearly represented in Bojarska’s study [13]. In our study, we could not evaluate the impact of sAPRIL levels on overall survival of CLL patients because the follow-up period was relatively short; we only lost 5 patients due to refractory disease during the follow-up. However, 4 of these 5 patients were in the sAPRIL high group.

sAPRIL levels were detected to be increased in our CLL population compared to healthy controls, paralleling the results of the previous reports. Moreover, we also demonstrated that sAPRIL levels in patients receiving chemotherapy were not different from those of the healthy controls. Thus, this finding indicates that sAPRIL levels were not useful for predicting the prognosis in patients undergoing treatment. Chemotherapy apparently led to a decrease in sAPRIL levels. In addition to this, we could demonstrate a negative correlation between the sAPRIL levels and hemoglobin as well as platelet counts. This finding was considered to be indirect evidence for an association between the leukemic cell burden and sAPRIL levels. 

Our study had some limitations. First of all, the correlation of sAPRIL levels with cytogenetic abnormalities and VH mutation could not be investigated due to the low number of patients with sufficient cytogenetic data. However, sAPRIL high and sAPRIL low groups were similar with regard to other prognostic factors. Second of all, the impact of sAPRIL levels on overall survival could not be determined due to the inadequate follow-up time. Last but not least, blood sampling times were heterogeneous—some blood samples were drawn at diagnosis, others during the course of the disease prior to or after a treatment episode. However, this limitation turned out to be a strength of the study, demonstrating the prognostic role of sAPRIL regardless of the collection time of serum samples in treatment-naïve CLL patients. The low number of treatment-naïve patients, the cross-sectional design of the study, and the relatively short duration of follow-up were other limitations.

In conclusion, sAPRIL levels are higher in CLL patients than in healthy controls, a finding that is in line with the current literature. However, this only holds true for treatment-naïve or treatment-free patients, not for those who are undergoing chemotherapy. Furthermore, sAPRIL levels seem to be correlated with leukemic cell burden. sAPRIL, a simple, promising blood test which can be measured by ELISA, will seemingly attain a place in the wide range of prognostic markers for CLL. Prospective large-scale randomized studies are required to validate and confirm the feasibility of the proposed cut-off level of 2.04 ng/mL as a predictor of time to treatment in treatment-naïve CLL patients. 

## Informed consent

This study was approved by the Ethics Committee of the Cerrahpaşa Medical Faculty (43458/2011) and was conducted in accordance with the 1964 Helsinki Declaration and its later amendments or comparable ethical standards.

Written informed consent was obtained from all patients and healthy controls.
